# Psychological stress documentation and subsequent risk of chronic inflammatory skin diseases: a large-scale real-world cohort study

**DOI:** 10.3389/fimmu.2026.1951015

**Published:** 2026-10-13

**Authors:** Philip Curman, Emelie Fischer, Jasmina Golaboska, Kaya Harms, Johanna Borowik, Julica Huckshold, Clara-Elisabeth Löscher, Marjam Nadjmabadi, Ayumi Maeda, Khashayar Pourrahimi, Sören Dräger, Hatim Kerniss, Jan Philipp Klein, Katja Bieber, Ralf J. Ludwig

**Affiliations:** 1 Dermatology and Venereology Division, Department of Medicine (Solna), Karolinska Institutet, Stockholm, Sweden; 2 Department of Medical Epidemiology and Biostatistics, Karolinska Institutet, Stockholm, Sweden; 3 Dermato-Venereology Clinic, Karolinska University Hospital, Stockholm, Sweden; 4 Lübeck Institute of Experimental Dermatology, University of Lübeck, Lübeck, Germany; 5 University of Lübeck, Lübeck, Germany; 6 QIMA Life Sciences, QIMA Monasterium GmbH, Münster, Germany; 7 Gifu University Graduate School of Medicine, Gifu, Japan; 8 Department of Dermatology, Allergology and Venerology, University Medical Center Schleswig-Holstein, Lübeck, Germany; 9 Department of Cardiology, University Heart Centre Frankfurt, University Hospital Frankfurt, Frankfurt/Main, Germany; 10 Department of Psychiatry and Psychotherapy , University Medical Center Schleswig-Holstein, Lübeck, Germany; 11 Institute and Comprehensive Center for Inflammation Medicine, University of Lübeck, Lübeck, Germany

**Keywords:** atopic dermatitis, chronic inflammatory skin diseases, cohort study, electronic health records, hidradenitis suppurativa, psoriasis, psychological stress, real-world evidence

## Abstract

**Background:**

Chronic inflammatory skin diseases (CISD) affect 20-25% of the population. Psychological stress is considered a modifiable risk factor, yet epidemiologic evidence remains inconsistent, partly due to challenges in capturing stress through clinical documentation.

**Objective:**

To investigate whether clinically documented psychological stress is associated with altered risk of incident CISDs.

**Methods:**

Retrospective cohort study. Individuals with ICD-coded psychological stress diagnoses propensity score-matched to controls on demographics, comorbidities, and healthcare utilization. Incident CISD risk was evaluated using Cox proportional hazards models over a median follow-up of 7.1 years. Robustness was assessed through seven sensitivity analyses, positive and negative control outcomes, model triangulation using multivariable Cox models, and quantitative bias analysis for exposure misclassification and empirical calibration of all primary estimates against 22 negative control outcomes.

**Results:**

Among 399, 615 matched pairs, ICD-coded stress showed no consistent association with incident atopic dermatitis, psoriasis vulgaris, vitiligo, lichen planus, alopecia areata, acne vulgaris, or rosacea. A modest association was observed for hidradenitis suppurativa (HR 1.20, 95% CI 1.13-1.27), which did not persist after empirical calibration (calibrated HR 1.17, 95% CI 1.00-1.37, calibrated p=0.049). Negative control outcomes revealed a small systematic upward shift, centred at HR 1.02. Bias analysis showed that incomplete documentation of stress among comparators could not account for the null associations.

**Limitations:**

Incomplete capture of psychological stress in electronic health records, potential diagnostic miscoding, residual unmeasured confounding, differential outcome ascertainment between cohorts, and lack of causal inference.

**Conclusion:**

After empirical calibration, ICD-coded stress diagnoses were not associated with incident CISD in US electronic health records, suggesting that stress documentation may not be a robust population-level predictor of CISD onset.

## 1 Introduction

Chronic non-communicable inflammatory skin diseases (CISDs) affect an estimated 20–25% of the population. Despite their heterogeneous clinical presentation, these diseases share core features of recurrent inflammation, and substantial effects on quality of life, work productivity, and health-care utilization (1). Excluding mortality, skin diseases collectively represent the fourth leading cause of disability worldwide (2). In sum, CISDs are a major contributor to global disease burden.

Psychological stress has long been considered a potentially modifiable risk factor for CISDs (3–5). Biologically, this hypothesis is plausible: Stress can affect neuroendocrine-immune pathways, barrier function, and inflammatory signaling (6, 7). However, epidemiologic evidence linking stress to the incident onset of CISDs remains inconsistent, largely because psychological stress is difficult to define, measure, and temporally anchor in observational studies (4, 8–10). Investigations have variously relied on self-reported perceived stress, stressful life events, occupational stress, validated stress scales, or psychiatric diagnoses as proxies, and many studies are retrospective, cross-sectional, or focused on disease exacerbation rather than first diagnosis. Moreover, recall bias is particularly problematic when patients retrospectively attribute disease onset to stressful events. This heterogeneity in exposure definition, study design, and temporal anchoring complicates interpretation (11).

Evidence across individual CISDs is heterogeneous. For example, atopic dermatitis illustrates the epidemiologic uncertainty of this association. On the one hand, longitudinal studies suggest that higher stress exposure may be associated with greater disease activity or higher odds of atopic dermatitis (12, 13), including adult-onset disease in some cohorts. On the other hand, effect sizes have generally been modest, and some studies have reported limited or no clear association, particularly when stress was measured in adulthood or when case numbers were small (8, 14). Psoriasis represents a second example in which the literature is extensive but methodologically mixed (4, 15): A systematic review and meta-analysis of 39 studies (32, 537 patients) found that recalled stressful events preceding psoriasis onset had a pooled OR of 3.4 (95% CI 1.8-6.4), but this was based primarily on retrospective studies with recall periods ranging from ≤9 months to ≥5 years. For diseases such as lichen planus or rosacea, the evidence base is sparse and consists largely of studies addressing psychological comorbidity or flare perception rather than temporally anchored incident disease risk (16, 17). Hidradenitis suppurativa is also frequently linked to stress and psychiatric burden, but much of that literature addresses disease activity, symptom worsening, or comorbidity rather than true incident onset (9, 18, 19).

Accordingly, a more rigorous assessment of whether clinically documented psychological stress is associated with altered risk of incident CISDs, rather than exacerbation of pre-existing disease, is needed. To address this question, we conducted a large-scale retrospective cohort study using electronic health record (EHR) data from the TriNetX research network (20). Individuals with ICD-coded psychological stress diagnoses were compared with propensity score-matched controls without recorded stress, and incident CISD risks were evaluated using Cox proportional hazards models. To strengthen robustness, we performed multiple sensitivity analyses, included positive and negative control outcomes, and triangulated findings using multivariable Cox models. We selected eight CISDs that are relatively common, arise from different pathogenic pathways, and impose at least moderate burden on affected patients: Atopic dermatitis, psoriasis vulgaris, vitiligo, lichen planus, alopecia areata, acne vulgaris, rosacea, and hidradenitis suppurativa.

By focusing specifically on incident disease rather than flares or worsening of pre-existing disease, this study aims to clarify the epidemiologic role of documented psychological stress across common and clinically important CISDs. Improved understanding of this association may help refine risk stratification, inform preventive strategies in clinical practice, and facilitate shared decision-making between clinicians and patients.

## 2 Materials and methods

### 2.1 Study design and database

A propensity score-matched (PSM) retrospective cohort study was conducted using the US Collaborative Network of the federated EHR TriNetX following established procedures (20, 21). Before the confirmatory analyses, scoping analyses were performed to assess the feasibility of the study design, specifically to determine whether the cohort definitions captured sufficient numbers of eligible EHRs, whether matching could be achieved adequately, and whether sufficient outcome events were available for meaningful analysis. Based on these feasibility assessments, the analytic framework, including cohort definitions, covariates, outcomes, and sensitivity analyses, was prespecified in full. A design freeze was then implemented, after which the confirmatory analyses were performed without further modification. Analyses were performed within the TriNetX platform using federated queries, with only aggregated results returned to investigators. The network was selected for its large EHR volume, detailed covariate documentation, and representation of the US health care-seeking population (22, 23). At the time of analysis (March 2026), the network contained approximately 110 million electronic health records (EHRs) from 67 healthcare organizations. The study evaluated the risk imposed by coded psychological stress on incident CISDs (**Figure 1**). Reporting followed the STROBE guidelines (24). The study was approved by the Swedish Ethical Review Authority (2025-03805-02); the analysis used de-identified secondary data with no interaction with human participants.

**Figure 1:**
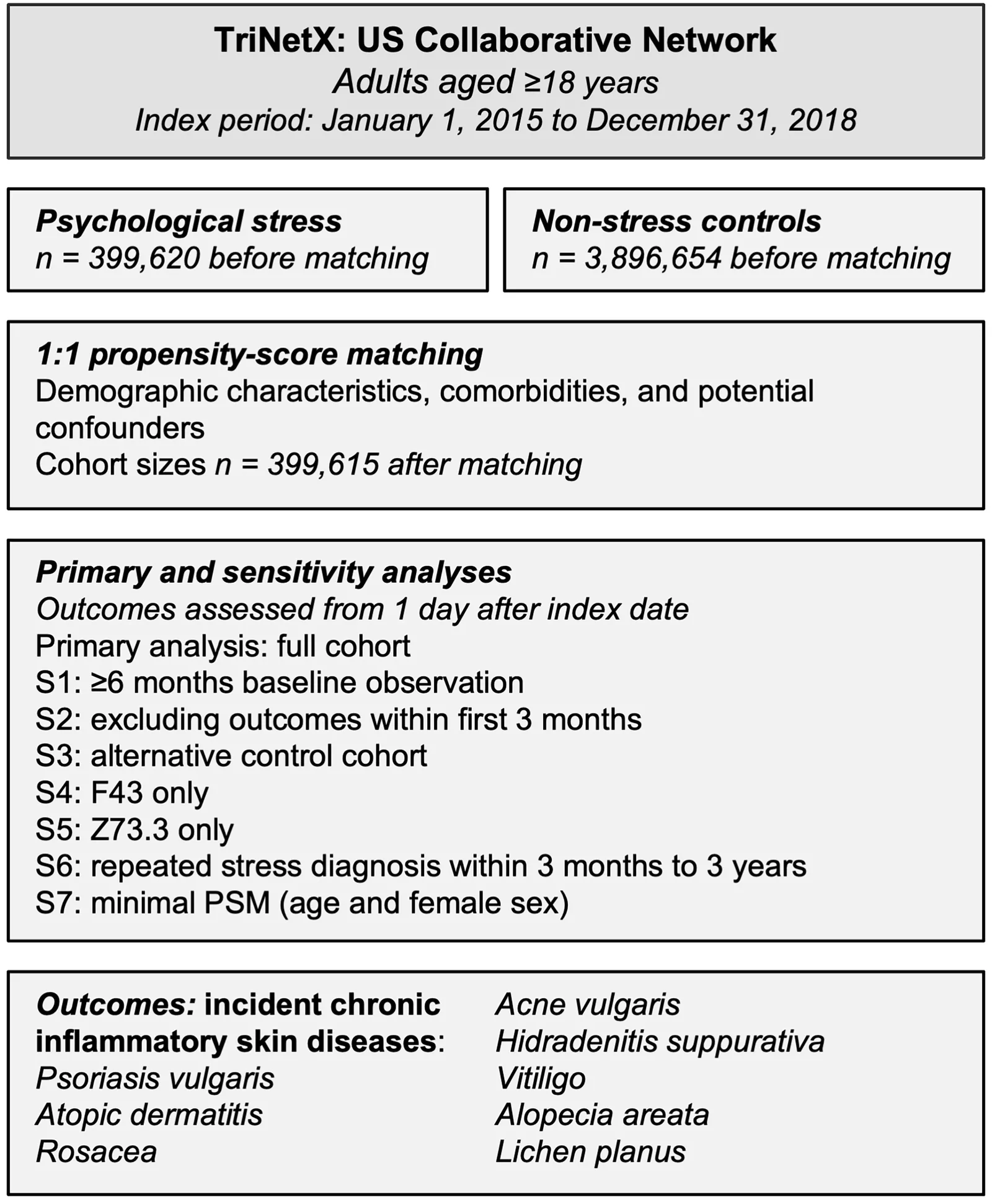
Study outline.

### 2.2 Study population and index events

Data were retrieved and analysed between March 9 and March 13, 2026. At the time of analysis, 67 health care organizations were queried and all responded, with 63–64 providers contributing patient data. The study population included adults (≥18 years). In the primary analysis, the index event for cases was defined as the first recorded diagnosis code for psychological stress (ICD-10-CM codes F43 [reaction to severe stress and adjustment disorders] or Z73.3 [stress, not elsewhere classified]) between January 1, 2015 and December 31, 2018. For controls, the index event was the first recorded general health examination (Z00) within the same period; controls were required to have no recorded stress diagnosis (F43 or Z73.3) prior to or on the index date to avoid immortal time bias. The index date corresponded to the first occurrence of the respective index event. Follow-up continued until the earliest of: Outcome occurrence, last recorded healthcare encounter, death, or end of study period. Detailed cohort and index event definitions are provided in **Supplementary Table 1**.

### 2.3 Outcomes

Outcomes were prespecified prior to data extraction. Incident CISDs were defined using ICD-10-CM codes recorded after the index date and included psoriasis vulgaris (L40.0), atopic dermatitis (L20), rosacea (L71), acne vulgaris (L70.0), hidradenitis suppurativa (L73.2), vitiligo (L80), alopecia areata (L63), and lichen planus (L43). Each outcome was analysed separately as a time-to-first-event endpoint. Predefined positive and negative control outcomes were analysed to assess the validity of the study design. Positive control outcomes included burns and corrosions of the external body surface (T20–T25) and depressive disorders (F32, F33), conditions for which an association with psychological stress has been documented (25, 26). Negative control outcomes included appendectomy (CPT codes 1014622, 44950, 44970) and presbycusis (H91.1), for which no causal association with psychological stress is expected (27, 28). Following peer review, 20 further negative control outcomes were added *post hoc*. All were selected as diseases, procedures or laboratory values with no hypothesised causal relationship to psychological stress: seborrhoeic keratosis (L82), melanocytic naevi (D22), ingrowing nail (L60.0), chloasma (L81.1), plantar wart (B07.0), benign lipomatous neoplasm of skin and subcutaneous tissue (D17.0–D17.3), excision of benign skin lesions (CPT 1003212) and destruction of benign lesions (CPT 17110); hallux valgus (M20.1), hallux rigidus (M20.2), ganglion of the wrist (M67.43), Achilles tendinitis (M76.6), lateral epicondylitis (M77.1) and palmar fascial fibromatosis [Dupuytren] (M72.0); deviated nasal septum (J34.2), acute maxillary sinusitis (J01.0), mucocele of the salivary gland (K11.6) and corneal degeneration (H18.4); extracorporeal shock wave lithotripsy (CPT 50590); and haemoglobin A1c ≥ 5.70% (TNX:9037). Eight of the 22 controls are cutaneous, although their ascertainment is only partly dermatological: benign skin lesions are also excised and destroyed in general surgery, plastic surgery and primary care, and ingrowing nails are frequently managed in podiatry. Outcomes were defined by the presence of the respective diagnosis or procedure code recorded in the EHR during follow-up beginning one day after the index event. Individuals with documented diagnoses of the respective outcome prior to the index event were excluded at data retrieval, as specified in the cohort construction criteria (**Supplementary Table 1**).

### 2.4 Covariates and propensity score matching

Covariates used for PSM are listed in **Supplementary Table 2**. A propensity score for each patient was estimated using logistic regression with exposure status as the dependent variable, implemented in the TriNetX analytics platform. Patients were matched 1:1 without replacement using a greedy nearest-neighbour algorithm with a caliper of 0.1 pooled standard deviations of the logit of the propensity score. Covariate balance before and after matching was assessed using standardized mean differences, with adequate balance defined as an absolute standardized mean difference <0.1. Missing covariate values were retained and treated as a separate category during PSM, which was performed for each comparison.

### 2.5 Primary and sensitivity analyses

In the primary analysis, patients with a first recorded diagnosis of psychological stress, defined as reaction to severe stress and adjustment disorders (F43) or stress, not elsewhere classified (Z73.3), were compared with non-stress controls identified by a general health examination encounter (Z00). The index date corresponded to the first occurrence of the respective index event between January 1, 2015 and December 31, 2018, and outcomes were assessed beginning one day after the index event. Seven pre-specified sensitivity analyses were performed to evaluate the robustness of the findings: (1) restriction to individuals with at least 6 months of baseline observation before the index event to ensure adequate capture of pre-existing diagnoses; (2) exclusion of outcomes occurring within the first 3 months after the index event to reduce potential reverse causation; (3) an alternative comparator cohort indexed on acute nasopharyngitis or impacted cerumen, conditions requiring a healthcare encounter but not preventive care seeking, to assess robustness of comparator selection; (4–5) restriction of exposure definitions separately to F43 and Z73.3 to evaluate potential heterogeneity in stress coding; (6) an alternative cohort construction requiring repeated stress diagnoses to capture more chronic exposure (note: this approach increases exposure specificity but introduces immortal time bias, as cohort qualification depended on post-index events); and (7) PSM using a minimal covariate set (age and sex only). Detailed cohort definitions for all analyses are provided in **Supplementary Table 1**. Healthcare contact (proxied by outpatient visits) during follow-up was quantified as the number of ambulatory encounters per person-year in the primary analysis.

### 2.6 Model triangulation

To test robustness across analytic approaches, multivariable Cox proportional hazards models were fitted in addition to the PSM analyses. Exposure status (coded psychological stress vs controls) was entered as the primary predictor. Models were adjusted for age, sex, ancestry (Black or African American), healthcare utilization, nicotine dependence, alcohol-related disorders, obesity, diabetes mellitus, and chronic lower respiratory diseases. Healthcare utilization was operationalized as a binary indicator of whether at least one encounter occurred during the baseline period, rather than as a count of encounters. Outcomes were assessed beginning one day after the index event, consistent with the primary analysis. Model triangulation was performed for all CISD outcomes, one positive control outcome (depression), and one negative control outcome (appendectomy). A reduced covariate set was used to maintain computational feasibility within the TriNetX platform while preserving identical cohort definitions to the primary analysis.

### 2.7 Quantitative bias analysis for exposure misclassification

To quantify the extent of exposure misclassification required to mask a true association, we performed probabilistic bias analysis on person-time data using the episensr package (v0.9.6) in R (29, 30). Because ICD-10 F43 (dominating the case group) is a diagnostic category with defined entry criteria assigned at a clinical encounter, false-positive coding was considered unlikely and the specificity of the exposure was fixed at 1.00. Misclassification was therefore modelled entirely as undocumented stress among comparators, denoted f, the fraction of comparator person-time contributed by individuals with clinically significant but uncoded stress. Sensitivity was assigned a trapezoidal prior distribution (0.75, 0.80, 0.90, 0.95), corresponding to f between 0.05 and 0.35, and 20, 000 Monte Carlo iterations were run for each outcome, and bias-adjusted estimates are reported with and without incorporation of random error. Estimates were taken from the primary PSM analysis, and person-time was reconstructed from the matched cohort sizes and the mean follow-up in each arm, with comparator person-time calibrated so that the crude incidence rate ratio equalled the hazard ratio of the primary analysis. We additionally computed, for each outcome, the value of f that would be required for the observed estimate to be compatible with true hazard ratios of 1.20, 1.30 and 1.50.

### 2.8 Statistical analysis

Baseline characteristics were compared using t-tests for continuous variables and z-tests for categorical variables. Time-to-event analyses were performed using the Kaplan–Meier method, with group differences assessed using the log-rank test. Hazard ratios (HR) with 95% confidence intervals (CI) were estimated using Cox proportional hazards models. The proportional hazards assumption was evaluated by examining Schoenfeld residuals. Bonferroni correction was applied across the eight CISD outcomes within each analysis (adjusted α = 0.00625); sensitivity analyses were evaluated separately. All of the above analyses were conducted within the TriNetX platform. Empirical calibration using the 22 negative controls (31) was performed using the method of Schuemie et al. (32, 33), implemented in the R package EmpiricalCalibration (34). A null distribution was fitted to the effect estimates of the 22 negative control outcomes by maximum likelihood, and calibrated p values and 95% confidence intervals were derived for each primary outcome by referring the observed estimate to that null. Calibration accounts for systematic error in addition to the random error captured by conventional intervals.

## 3 Results

### 3.1 Cohort description and healthcare encounters during follow-up

A total of 399, 620 adults with documented psychological stress and 3, 896, 654 controls were identified before PSM. After matching, 399, 615 cases were successfully paired with an equal number of controls. Before matching, several baseline characteristics showed notable imbalance. For example, the proportion of female participants was higher among cases than controls (62.7% vs. 54.5%; SMD = 0.168), and nicotine dependence was more prevalent among cases (11.6% vs. 5.2%; SMD = 0.230). After matching, these differences were attenuated: sex distribution was balanced (62.7% vs. 62.7%; SMD = 0.0004), and nicotine dependence prevalence was similar (11.6% vs. 11.8%; SMD = 0.007). All covariates demonstrated minimal standardized differences after matching, indicating well-balanced groups (**Table 1**; **Supplementary Table 2**). Follow-up is reported in **Supplementary Table 2**.

**Table 1:** Baseline characteristics of individuals with documented psychological stress and matched controls.

Covariate	Before matching			After matching		
	Cases	Controls	Std diff.	Cases	Controls	Std diff.
n	399, 620	3, 896, 654	NA	399, 615	399, 615	NA
Follow-up, days (median, interquartile range)	2, 590 (1, 940)	2, 707 (1, 771)	NA	2, 590 (1, 940)	2, 729 (1, 692)	NA
Age at Index, years	45.4 ± 16.7	47.7 ± 17.2	0.1343	45.4 ± 16.7	45.6 ± 16.7	0.0070
Female, %	62.7	54.5	0.1677	62.7	62.7	0.0004
Male, %	37.3	44.5	0.1486	37.3	36.9	0.0075
Asian, %	3.1	4.2	0.0613	3.1	2.9	0.0076
Black or African American, %	15.4	13.0	0.0704	15.4	15.6	0.0065
Hispanic or Latino, %	8.0	5.6	0.0953	8.0	7.9	0.0033
White, %	70.9	70.8	0.0023	70.9	71.0	0.0017
Nicotine dependence, %	11.6	5.2	0.2302	11.6	11.8	0.0073
Personal history of nicotine dependence, %	6.0	3.7	0.1038	6.0	6.1	0.0039
Alcohol related disorders, %	3.7	1.4	0.1507	3.7	3.8	0.0039
Persons with potential health hazards related to socioeconomic and psychosocial circumstances, %	3.6	0.9	0.1798	3.6	3.4	0.0094
Family history of diseases of the skin and subcutaneous tissue, %	0.0	0.0	0.0009	0.0	0.0	0.0044
Overweight and obesity, %	13.3	9.0	0.1370	13.3	13.5	0.0046
Diabetes mellitus, %	10.6	8.3	0.0780	10.6	10.6	0.0012
Chronic lower respiratory diseases, %	14.3	9.5	0.1484	14.3	14.3	0.0014
Chronic kidney disease, %	3.5	2.5	0.0594	3.5	3.5	0.0001
Hypertensive diseases, %	25.3	22.7	0.0624	25.3	25.5	0.0040
Malignant neoplasms of breast, %	1.7	1.2	0.0411	1.7	1.7	0.0012
Malignant melanoma of skin, %	0.4	0.4	0.0017	0.4	0.3	0.0066
Malignant neoplasm of prostate, %	0.6	0.8	0.0210	0.6	0.6	0.0025
Malignant neoplasm of bronchus and lung, %	0.5	0.3	0.0383	0.5	0.5	0.0030
Malignant neoplasms of digestive organs, %	1.0	0.6	0.0474	1.0	1.0	0.0018

Baseline demographic and clinical characteristics of individuals with documented psychological stress (cases) and controls without recorded stress before and after propensity score matching (PSM). Continuous variables are presented as mean ± standard deviation or median (interquartile range), and categorical variables as percentages. Standardized differences (Std diff.) were used to assess covariate balance between groups. Follow-up time is reported in days from index date to last recorded encounter or end of follow-up. Abbreviations: IQR, interquartile range.

To evaluate whether differential healthcare seeking behaviour accounts for any differences, we compared healthcare contact during follow in the primary analysis. Patients with a documented stress diagnosis had substantially more clinical contact during follow-up than matched comparators. At least one ambulatory encounter was recorded for 89.3% of exposed individuals and 88.4% of comparators. However, ambulatory visit rates were 11.3 per person-year in the exposed cohort and 8.7 per person-year among comparators. This differential healthcare encounters during follow-up bias estimates away from the null in the direction of apparent harm, but elevated estimates may be biased by differential health care utilization.

### 3.2 Behaviour of control outcomes

To assess internal validity and residual confounding, two positive and four negative control outcomes were evaluated. Depression and burns/corrosions of external body surface served as positive controls, given their established associations with psychological stress (25, 26). Both demonstrated significant associations in the expected direction (depression: HR 2.38, 95% CI 2.35–2.40, p < 0.0001; burns: HR 1.56, 95% CI 1.49–1.63, p < 0.0001). Presbycusis and appendectomy served as negative controls, as neither is plausibly linked to psychological stress (27, 28). As expected, neither outcome was associated with coded stress (presbycusis: HR 1.10, 95% CI 0.98–1.23, p = 0.093; appendectomy: HR 1.06, 95% CI 0.99–1.14, p = 0.107). Both dermatological negative control outcomes (seborrhoeic keratosis and melanocytic naevi) were recorded significantly less frequently in the exposed cohort (seborrhoeic keratosis: HR 0.90, 95% CI 0.89–0.91, p < 0.0001; melanocytic naevi: HR 0.83, 95% CI 0.82–0.85, p < 0.0001), indicating that dermatologic surveillance in this design favoured the comparator cohort rather than the exposed cohort. These findings were directionally consistent in multivariable Cox models (**Figures 2**, **3**; **Supplementary Tables 2**, **3**). The negative controls behaved differently under the two comparators: presbycusis lay marginally above the null against general-examination controls (HR 1.10) and well below it against the acute-care comparator of S3 (HR 0.64, 95% CI 0.58–0.71), consistent with preferential ascertainment of hearing loss among individuals presenting with impacted cerumen, whereas appendectomy was null under both (1.06 and 1.04). Comparator choice therefore influences which outcomes are preferentially ascertained, and the control outcomes constrain rather than simply confirm the analytic framework.

**Figure 2:**
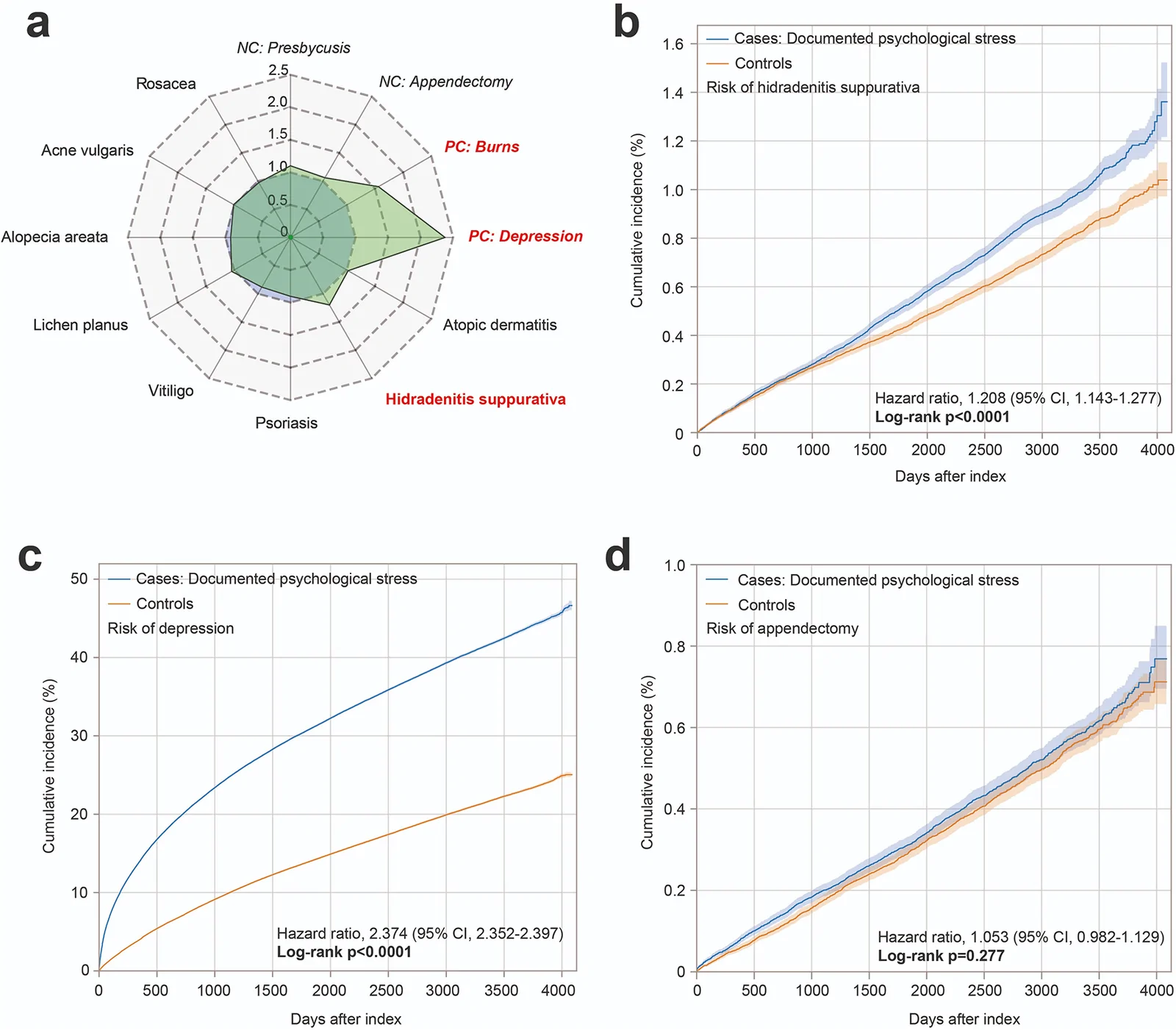
Hazard ratios for incident outcomes following documented psychological stress. **(A)** Radar plot showing hazard ratios (HRs) for incident dermatologic and control outcomes in the primary analysis. Values represent HR estimates from Cox proportional hazards models comparing individuals with documented psychological stress to matched controls. Negative control outcomes (presbycusis, appendectomy) and positive control outcomes (burns and corrosions of external body surface, depression) are displayed in italics. Red indicates statistical significance after Bonferroni correction. Most chronic inflammatory skin diseases showed no increased risk following documented stress; a modest association was observed for hidradenitis suppurativa. Shading indicates HR ≤1 (light blue) or >1 (light gray). **(B–D)** Kaplan–Meier curves for **(B)** hidradenitis suppurativa, **(C)** depression (positive control), and **(D)** appendectomy (negative control). Shaded areas represent 95% confidence intervals.

**Figure 3:**
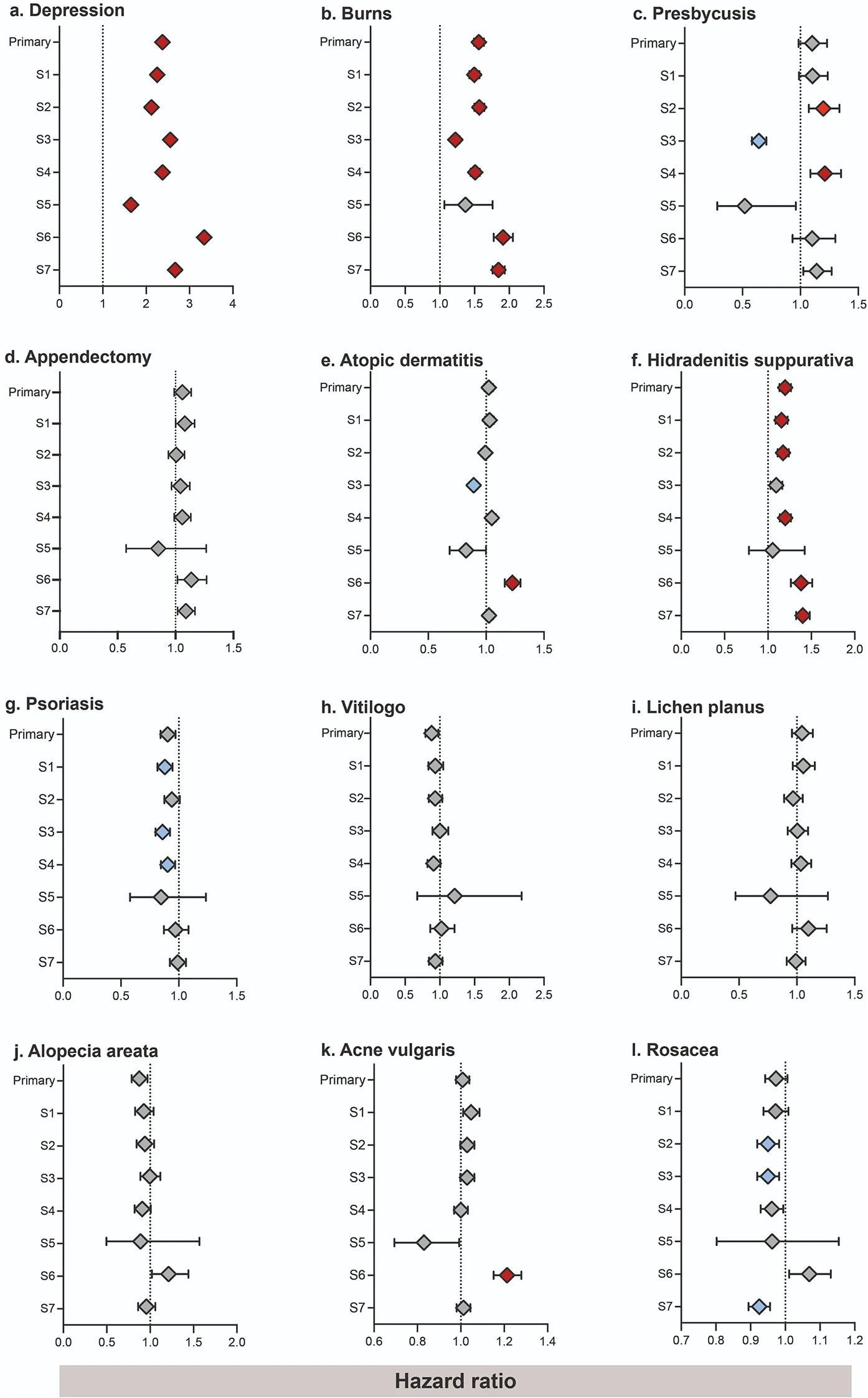
Hazard ratios for incident chronic inflammatory skin diseases and control outcomes across primary and sensitivity analyses. Forest plots showing HRs with 95% confidence intervals (horizontal bars) for incident outcomes comparing individuals with documented psychological stress to matched controls. Panels show **(A)** depression, **(B)** burns and corrosions of external body surface, **(C)** presbycusis, **(D)** appendectomy, **(E)** atopic dermatitis, **(F)** hidradenitis suppurativa, **(G)** psoriasis vulgaris, **(H)** vitiligo, **(I)** lichen planus, **(J)** alopecia areata, **(K)** acne vulgaris and **(L)** rosacea. Diamonds indicate point estimates for the primary analysis and sensitivity analyses (S1–S7). Positive controls **(A, B)** and negative controls **(C, D)** assessed analytical validity. Color coding: grey = non-significant; red = significantly increased risk (HR >1); blue = significantly decreased risk (HR <1). HRs and 95% CIs are presented without multiplicity adjustment; significance was determined using Bonferroni-corrected thresholds (α = 0.00625 for CISD outcomes).

### 3.3 Documented psychological stress and risk of incident CISDs

For seven of the eight CISDs evaluated, documented psychological stress was not associated with increased risk of incident disease. Results were largely consistent across sensitivity analyses and model triangulation (**Figures 2**, **3**; **Supplementary Table 2**). Atopic dermatitis showed no significant association with stress documentation (HR 1.02, 95% CI 0.99–1.06, p = 0.23) – except in S3 where the estimate under the alternative comparator was modestly inverse (HR 0.89, 95% CI 0.86–0.93). Similarly, rosacea demonstrated no consistent association; although one sensitivity analysis yielded a modest inverse estimate (S2: HR 0.95, 95% CI 0.92–0.98, p = 0.0025), this was not replicated across other analyses. Multivariable Cox models yielded comparable estimates across all CISD outcomes (**Supplementary Table 3**).

### 3.4 Undocumented stress in the comparator cohort is unlikely to account for the null associations

Because comparators were defined by the absence of a stress code rather than by the absence of stress, some will have experienced clinically significant stress without documentation, diluting the observed contrast. Quantifying this dilution shows that it cannot generate the pattern observed (**Supplementary Table 4**): Misclassification of this direction would attenuate an association towards the null but cannot carry an estimate across it. The null associations obtained for psoriasis vulgaris (HR 0.90), alopecia areata (0.92), vitiligo (0.88) and rosacea (0.97) are therefore incompatible with any elevated true hazard ratio. For the four estimates above unity, compatibility with a true hazard ratio of 1.30 would require that 91% (atopic dermatitis), 97% (acne vulgaris), 82% (lichen planus) or 29% (hidradenitis suppurativa) of comparators were under clinically significant stress without ever receiving a diagnostic code (**Supplementary Table 4**). Conversely, a true hazard ratio of 1.30 would still have been observed as 1.21 had a quarter of comparators been silently stressed, and as 1.13 had half been. In probabilistic bias analysis assuming perfect specificity and 5–35% undocumented stress among comparators, bias-adjusted hazard ratios remained null for atopic dermatitis (1.03, 95% simulation interval 0.99–1.06), lichen planus (1.05, 0.96–1.15), acne vulgaris (1.01, 0.98–1.04), rosacea (0.97, 0.94–1.00), alopecia areata (0.90, 0.82–1.00), psoriasis vulgaris (0.89, 0.82–0.95) and vitiligo (0.86, 0.77–0.96), and increased modestly for hidradenitis suppurativa (1.25, 1.17–1.34) (**Supplementary Table 4**).

### 3.5 Documented psychological stress and risk of incident hidradenitis suppurativa

In contrast to the null findings for other CISDs, documented psychological stress was associated with a modest increase in incident hidradenitis suppurativa risk (**Figures 2**, **3**; **Supplementary Tables 2**, **3**). In the primary analysis, hidradenitis suppurativa was documented in 0.66% of cases versus 0.60% of controls (HR 1.20, 95% CI 1.13–1.27, p < 0.0001). Accounting for differential observation time between the cohorts, Kaplan–Meier cumulative incidence at the end of follow-up was 1.36% in individuals with documented stress and 1.04% in comparators, an absolute difference of 0.32%. Hazard ratios exceeded 1.0 across all sensitivity analyses, with six of seven reaching statistical significance. The strongest association was observed using a chronic stress exposure definition (0.87% vs. 0.65%; HR 1.38, 95% CI 1.26–1.51, p < 0.0001). Multivariable Cox models yielded comparable estimates (HR 1.19, 95% CI 1.11–1.27, p < 0.0001). Overall, coded psychological stress demonstrated a modest but consistent association with increased HS risk in uncalibrated analyses (see section 3.6).

### 3.6 After empirical calibration, documented stress was not associated with incident hidradenitis suppurativa

To quantify residual systematic error, the effect estimates of the 22 negative control outcomes were used to fit an empirical null distribution, against which the p values and confidence intervals of all primary estimates were calibrated (31). The empirical null was centred at HR 1.023 (95% CI 0.989–1.058), with 95% of estimates for outcomes having no true effect expected to fall between HR 0.88 and 1.18. This corresponds to an expected absolute systematic error of 0.062 on the log scale, equivalent to a typical displacement of approximately 6% in either direction (**Figure 4A**). Estimates in this design are therefore displaced slightly away from the null in the direction of apparent harm. The control panel also provides a direct measure of the design’s type-1 error rate. Ten of the 22 negative controls were significant at the prespecified threshold (α = 0.00625) before calibration and none afterwards. After calibration, none of the eight CISD outcomes, including hidradenitis suppurativa met the prespecified Bonferroni-corrected threshold (**Figure 4B**). Atopic dermatitis calibrated to HR 1.00 (95% CI 0.86–1.16), lichen planus to 1.02 (0.86–1.21), acne vulgaris to 0.99 (0.85–1.14), rosacea to 0.95 (0.82–1.11), alopecia areata to 0.90 (0.75–1.07), psoriasis vulgaris to 0.88 (0.75–1.04) and vitiligo to 0.86 (0.72–1.03). The hidradenitis suppurativa estimate attenuated from 1.20 (1.13–1.27) to 1.17 (1.00–1.37), with a calibrated p of 0.049. Notably, calibration attenuated but did not abolish the positive control associations: depression from 2.38 (2.35–2.40) to 2.32 (2.01–2.69) and burns and corrosions from 1.56 (1.49–1.63) to 1.53 (1.31–1.78).

**Figure 4:**
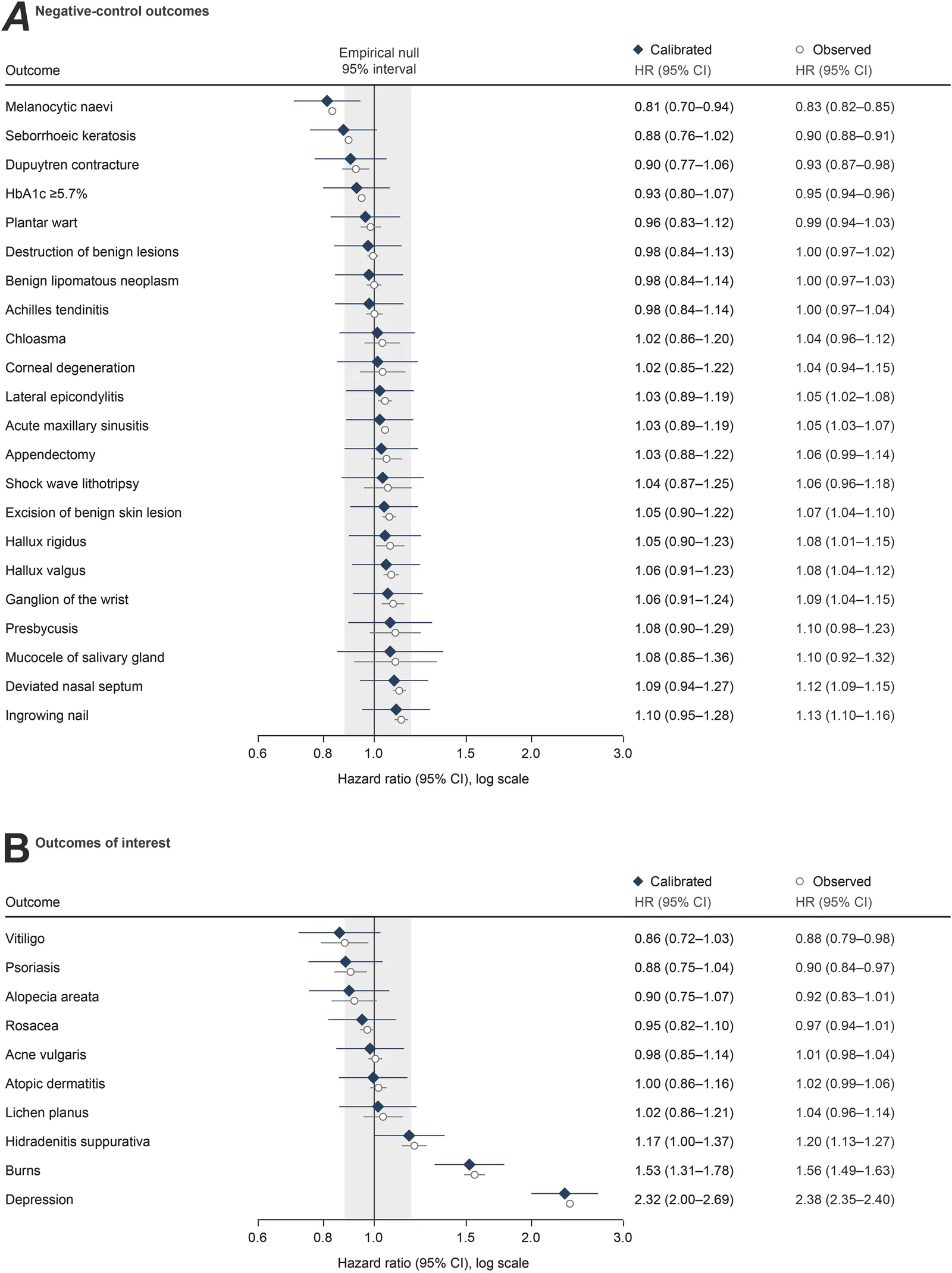
Empirical calibration of effect estimates against 22 negative control outcomes. **(A)** Hazard ratios for the 22 negative control outcomes, ordered from lowest to highest observed estimate. The black dotted line marks unity, the red dashed line the centre of the empirical null (HR 1.023), and the red shaded area its 95% limits (HR 0.88–1.18). **(B)** Hazard ratios for the eight chronic inflammatory skin disease outcomes and the two positive control outcomes (burns and corrosions of the external body surface; depressive disorders), ordered in the same way. Here the red dashed line marks unity, since calibration removes the systematic shift and calibrated estimates are therefore referred to unity directly. In both panels, grey circles show the observed estimates and black diamonds the estimates after empirical calibration; horizontal bars are 95% confidence intervals.

## 4 Discussion

In this large real-world cohort study, clinically documented psychological stress was not consistently associated with increased risk of incident CISDs. Across primary and sensitivity analyses, as well as model triangulation, documented stress did not alter the risk of incident atopic dermatitis, psoriasis vulgaris, vitiligo, lichen planus, alopecia areata, acne vulgaris, or rosacea. The exception was hidradenitis suppurativa, for which a modest but consistent increase in risk was observed. Although the effect size was small, the association remained directionally stable across sensitivity analyses but did not survive empirical calibration (calibrated HR 1.17, 95% CI 1.00–1.37). Importantly, the analytical framework was constrained rather than confirmed by the control outcomes: the positive controls remained strongly associated after calibration, whereas ten of the 22 negative controls were significant before calibration, indicating a small systematic shift in the direction of apparent harm. Collectively, these findings suggest that coded psychological stress may not be a major population-level risk factor for CISD onset, and that the apparent exception for hidradenitis suppurativa cannot be reliably separated from residual systematic error in this design.

Psychological stress has long been considered clinically relevant in inflammatory skin diseases. However, much of the existing literature focuses on disease exacerbation, symptom severity, or patient-reported outcomes rather than incident disease onset. Evidence specifically linking stress to first manifestation of CISDs remains sparse (4, 35–38). Regarding HS, a population-based Danish registry study examining disease trajectories found that mental and behavioral disorders, including stress-related conditions, occurred more frequently before HS diagnosis (9). These findings complement the present results by situating documented stress within broader HS disease trajectories and supporting a modest association between coded psychological stress and HS onset. Interpretation of this association is complicated by the diagnostic trajectory of HS itself. Real-world data from six countries indicate a symptom-to-diagnosis interval of seven to ten years (39), so a substantial proportion of individuals recorded as incident HS during follow-up will already have been symptomatic before the index stress code was entered. Distress arising from undiagnosed disease would therefore precede rather than follow the recorded exposure, and reverse causation is a leading explanation for the association observed. The strongest estimate was obtained when exposure was defined as repeatedly coded (“chronic”) stress; that analysis is subject to immortal time bias, is reported as exploratory, and we do not interpret it as evidence of a dose–response relationship.

The null findings for atopic dermatitis, psoriasis vulgaris, and other CISDs require contextualisation against a literature that has largely addressed a different question. Much of the evidence linking psychological factors to these diseases concerns disease activity, symptom severity, or health-related quality of life in patients with established disease, not incident onset in disease-free individuals. Studies reporting associations between stress and atopic dermatitis or psoriasis have often relied on retrospective recall of pre-onset stress, cross-sectional designs, or outcomes reflecting flares rather than first diagnosis. The present findings do not contradict the well-documented psychosocial burden of these diseases; rather, they suggest that while stress may modulate disease course and patient-reported outcomes, clinically documented stress may not function as a consistent population-level trigger for disease initiation. Much of the psychological distress surrounding vitiligo appears to arise from others’ responses to visible depigmentation rather than from the disease process itself (40, 41). Distress of that origin cannot arise before the depigmentation is visible: it follows the appearance of disease, and usually its diagnosis, rather than preceding it. Our design examined only the period before diagnosis, in individuals still free of vitiligo, and would therefore not capture the distress that patients later describe.

This study has several strengths. First, the large real-world population enabled evaluation of relatively uncommon outcomes such as HS. Second, multiple methodological safeguards were implemented, including PSM, sensitivity analyses, and model triangulation. Third, the inclusion of an expanded panel of 22 negative control outcomes allowed the direction and magnitude of residual systematic error to be estimated and corrected for by empirical calibration. Fourth, comparator choice determined which outcomes were preferentially ascertained, as the divergent behaviour of presbycusis across our two comparators illustrates. That estimates for chronic inflammatory skin disease were nonetheless stable across comparators with opposing ascertainment profiles argues against detection bias as the explanation for our null findings.

However, several limitations warrant consideration. First, ICD-coded psychological stress reflects clinical documentation rather than true stress exposure, and is shaped by healthcare-seeking behavior, clinician coding practice, psychiatric context, and socioeconomic factors; the exposure should therefore be interpreted as documented rather than experienced stress, and is likely undercoded. However, it is unlikely to account for our null findings. Because such misclassification can only attenuate an association towards the null, the inverse estimates obtained for four of the eight diseases studied are incompatible with any elevated true risk, and reproducing the remaining null estimates from a true hazard ratio of 1.30 would require that the majority of comparators were under clinically significant but undocumented stress. Second, the retrospective design precludes causal inference. Third, reliance on ICD coding introduces potential misclassification due to coding variability or incomplete documentation. Fourth, incident status was defined by the absence of a recorded prior diagnosis rather than a true absence of prior disease. Relating to acne vulgaris: much L70.0 coding in adults reflects persistent adolescent-onset disease first entered into the record in adulthood, particularly where adolescent care predates the available data. The acne estimate should therefore be read as pertaining to the first adult recording of acne. Fifth, the exposure was carried almost entirely by F43: restricting the index event to F43 alone (S4) yielded 418, 741 matched pairs, whereas restricting it to Z73.3 alone (S5) yielded 11, 312. Hence, the results and conclusions are almost exclusively based on the F43 diagnostic code. Sixth, residual confounding cannot be fully excluded despite PSM, control outcomes, and multivariable adjustment. Seventh, violations of the proportional hazards assumption occurred in some analyses, which may affect interpretation of hazard ratios. Eighth, healthcare contact during follow-up was higher among individuals with documented stress. This potentially inflates estimates in the direction of apparent harm and so cannot explain our null and (inconsistent) inverse associations. By contrast, the estimate for hidradenitis suppurativa, where we documented an increased risk following coded stress, may be confounded by differential healthcare utilization. Ascertainment of skin disease differed between the cohorts by pathway rather than by volume. Incidentally detected lesions were recorded less often in individuals with documented stress, whereas conditions that patients bring to attention themselves lay at or above the null. Skin-specific surveillance therefore favoured the comparator cohort, while complaint-driven ascertainment favoured the exposed cohort. This does not exclude the possibility that a higher rate of clinical encounters brought symptomatic disease to attention earlier, and it is directly relevant to hidradenitis suppurativa, which is ascertained almost entirely through symptomatic presentation. Finally, all data derive from the US Collaborative Network of TriNetX. Health systems, access to care, help-seeking behaviour and - most consequentially for a study whose exposure is a diagnostic code - the practice of documenting psychological distress differ substantially between countries. That the exposure here was carried almost entirely by F43, with Z73.3 contributing few individuals, plausibly reflects US coding conventions rather than the underlying distribution of stress. These findings should therefore be read as applying to the US healthcare context and may not generalise to other settings, including the European health systems in which several of the authors work. Incomplete follow-up and variation in care patterns across institutions may further affect generalizability.

In conclusion, ICD-coded psychological stress was not consistently associated with increased risk of incident CISDs in this large real-world analysis. The single elevated estimate, for hidradenitis suppurativa, did not survive empirical calibration. Because the exposure indexes clinical documentation rather than experienced stress, these findings constrain the predictive value of stress documentation in routine care rather than the role of stress itself. Within these limitations, our data suggest that ICD-coded psychological stress is not a major population-level predictor of incident CISD.

## Data Availability

The raw data supporting the conclusions of this article will be made available by the authors, without undue reservation.
