# Supplementary material for: Psychological stress documentation and subsequent risk of chronic inflammatory skin diseases: a large-scale real-world cohort study
Source: Front Immunol. 2026 Oct 13;17:1951015. doi: 10.3389/fimmu.2026.1951015 (PMC13710232; doi:10.3389/fimmu.2026.1951015)
Supplement: Supplementary file 1 [file Table1.docx]

**Supplement Table 1.** Cohort construction, index event definitions, and temporal rules for the primary and sensitivity analyses.

| **Primary analysis, S1 (6 months MBW), and S7 (minimal PSM)** | | | | |
| --- | --- | --- | --- | --- |
| **Cohort** | **Query group** | **Definition** | **Codes** | **Temporal rule** |
| **Psychological stress (cases)** | Group 1 | Index event: first recorded diagnosis of psychological stress | ICD-10-CM F43 – Reaction to severe stress and adjustment disorders; Z73.3 – Stress, not elsewhere classified | Event between 1 Jan 2015 and 31 Dec 2018; age ≥18 years |
|  | Group 2A | Index event replication used for cohort construction | Same as Group 1 | Same date window |
|  | Group 2B | Exclusion criteria prior to cohort entry | Burns/corrosions (T20–T25); Presbycusis (H91.1); Appendectomy (CPT 1014622, 44950, 44970); Rosacea (L71); Psoriasis vulgaris (L40.0); Atopic dermatitis (L20); Acne vulgaris (L70.0); Hidradenitis suppurativa (L73.2); Vitiligo (L80); Alopecia areata (L63); Lichen planus (L43); Depressive episode (F32); Major depressive disorder (F33) | Any instance of Group 2B occurred on or before the first instance of Group 2A |
| **Non-stress controls** | Group 1A | Index event: general health examination encounter | ICD-10-CM Z00 – Encounter for general examination without complaint or suspected diagnosis | Event between 1 Jan 2015 and 31 Dec 2018; age ≥18 years |
|  | Group 1B | Exclusion of psychological stress diagnoses prior to index | ICD-10-CM F43, Z73.3 | Any instance of Group 1B occurred on or before the first instance of Group 1A |
|  | Group 2A | Index event replication used for cohort construction | Same as Group 1A | Same date window |
|  | Group 2B | Exclusion criteria prior to cohort entry | Same exclusions as listed for cases | Any instance of Group 2B occurred on or before the first instance of Group 2A |

| **S2 (3 months lag-period)** | | | | |
| --- | --- | --- | --- | --- |
| **Cohort** | **Query group** | **Definition** | **Codes** | **Temporal rule** |
| **Psychological stress (cases)** | Group 1 | Index event: first recorded diagnosis of psychological stress | ICD-10-CM F43 – Reaction to severe stress and adjustment disorders; Z73.3 – Stress, not elsewhere classified | Event between 1 Jan 2015 and 31 Dec 2018; age ≥18 years |
|  | Group 2A | Index event replication used for cohort construction | Same as Group 1 | Same date window |
|  | Group 2B | Exclusion criteria prior to cohort entry | Burns/corrosions (T20–T25); Presbycusis (H91.1); Appendectomy (CPT 1014622, 44950, 44970); Rosacea (L71); Psoriasis vulgaris (L40.0); Atopic dermatitis (L20); Acne vulgaris (L70.0); Hidradenitis suppurativa (L73.2); Vitiligo (L80); Alopecia areata (L63); Lichen planus (L43); Depressive episode (F32); Major depressive disorder (F33) | Any instance of Group 2B occurred on or before the first instance of Group 2A |
|  | Group 3A | Index event replication used for baseline requirement | Same as Group 1 | Same date window |
|  | Group 3B | Minimum baseline observation | Visit | Any instance of Group 3B occurred ≥6 months before the first instance of Group 3A |
| **Non-stress controls** | Group 1A | Index event: general health examination encounter | ICD-10-CM Z00 – Encounter for general examination without complaint or suspected diagnosis | Event between 1 Jan 2015 and 31 Dec 2018; age ≥18 years |
|  | Group 1B | Exclusion of psychological stress diagnoses prior to index | ICD-10-CM F43, Z73.3 | Any instance of Group 1B occurred on or before the first instance of Group 1A |
|  | Group 2A | Index event replication used for cohort construction | Same as Group 1A | Same date window |
|  | Group 2B | Exclusion criteria prior to cohort entry | Same exclusions as listed for cases | Any instance of Group 2B occurred on or before the first instance of Group 2A |
|  | Group 3A | Index event replication used for baseline requirement | Same as Group 1A | Any instance of Group 3B occurred ≥6 months before the first instance of Group 3A |
|  | Group 3B | Minimum baseline observation | Visit |  |

| **S3 (alternative definition of controls)** | | | | |
| --- | --- | --- | --- | --- |
| **Cohort** | **Query group** | **Definition** | **Codes** | **Temporal rule** |
| **Psychological stress (cases)** | Group 1 | Index event: first recorded diagnosis of psychological stress | ICD-10-CM F43 – Reaction to severe stress and adjustment disorders; Z73.3 – Stress, not elsewhere classified | Event between 1 Jan 2015 and 31 Dec 2018; age ≥18 years |
|  | Group 2A | Index event replication used for cohort construction | Same as Group 1 | Same date window |
|  | Group 2B | Exclusion criteria prior to cohort entry | Burns/corrosions (T20–T25); Presbycusis (H91.1); Appendectomy (CPT 1014622, 44950, 44970); Rosacea (L71); Psoriasis vulgaris (L40.0); Atopic dermatitis (L20); Acne vulgaris (L70.0); Hidradenitis suppurativa (L73.2); Vitiligo (L80); Alopecia areata (L63); Lichen planus (L43); Depressive episode (F32); Major depressive disorder (F33) | Any instance of Group 2B occurred on or before the first instance of Group 2A |
| **Non-stress controls** | Group 1A | Acute nasopharyngitis or impacted cerumen | ICD-10-CM J00 – Acute nasopharyngitis (common cold); H61.2 – Impacted cerumen | Event between 1 Jan 2015 and 31 Dec 2018; age ≥18 years |
|  | Group 1B | Exclusion of psychological stress diagnoses prior to index | ICD-10-CM F43 – Reaction to severe stress and adjustment disorders; Z73.3 – Stress, not elsewhere classified | Any instance of Group 1B occurred on or before the first instance of Group 1A |
|  | Group 2A | Index event replication used for cohort construction | Same as Group 1A | Same date window |
|  | Group 2B | Exclusion criteria prior to cohort entry | Burns/corrosions (T20–T25); Presbycusis (H91.1); Appendectomy (CPT 1014622, 44950, 44970); Rosacea (L71); Psoriasis vulgaris (L40.0); Atopic dermatitis (L20); Acne vulgaris (L70.0); Hidradenitis suppurativa (L73.2); Vitiligo (L80); Alopecia areata (L63); Lichen planus (L43); Depressive episode (F32); Major depressive disorder (F33) | Any instance of Group 2B occurred on or before the first instance of Group 2A |

| **S4 (alternative index definition; ICD-10-CM:F43 only)** | | | | |
| --- | --- | --- | --- | --- |
| **Cohort** | **Query group** | **Definition** | **Codes** | **Temporal rule** |
| **Psychological stress (cases)** | Group 1 | Index event: first recorded diagnosis of psychological stress | ICD-10-CM F43 – Reaction to severe stress and adjustment disorders | Event between 1 Jan 2015 and 31 Dec 2018; age ≥18 years |
|  | Group 2A | Index event replication used for cohort construction | Same as Group 1 | Same date window |
|  | Group 2B | Exclusion criteria prior to cohort entry | Burns/corrosions (T20–T25); Presbycusis (H91.1); Appendectomy (CPT 1014622, 44950, 44970); Rosacea (L71); Psoriasis vulgaris (L40.0); Atopic dermatitis (L20); Acne vulgaris (L70.0); Hidradenitis suppurativa (L73.2); Vitiligo (L80); Alopecia areata (L63); Lichen planus (L43); Depressive episode (F32); Major depressive disorder (F33) | Any instance of Group 2B occurred on or before the first instance of Group 2A |
| **Non-stress controls** | Group 1A | Index event: general health examination encounter | ICD-10-CM Z00 – Encounter for general examination without complaint or suspected diagnosis | Event between 1 Jan 2015 and 31 Dec 2018; age ≥18 years |
|  | Group 1B | Exclusion of psychological stress diagnoses prior to index | ICD-10-CM F43, Z73.3 | Any instance of Group 1B occurred on or before the first instance of Group 1A |
|  | Group 2A | Index event replication used for cohort construction | Same as Group 1A | Same date window |
|  | Group 2B | Exclusion criteria prior to cohort entry | Same exclusions as listed for cases | Any instance of Group 2B occurred on or before the first instance of Group 2A |

| **S5 (alternative index definition; ICD-10-CM:Z73.3 only)** | | | | |
| --- | --- | --- | --- | --- |
| **Cohort** | **Query group** | **Definition** | **Codes** | **Temporal rule** |
| **Psychological stress (cases)** | Group 1 | Index event: first recorded diagnosis of psychological stress | Z73.3 – Stress, not elsewhere classified | Event between 1 Jan 2015 and 31 Dec 2018; age ≥18 years |
|  | Group 2A | Index event replication used for cohort construction | Same as Group 1 | Same date window |
|  | Group 2B | Exclusion criteria prior to cohort entry | Burns/corrosions (T20–T25); Presbycusis (H91.1); Appendectomy (CPT 1014622, 44950, 44970); Rosacea (L71); Psoriasis vulgaris (L40.0); Atopic dermatitis (L20); Acne vulgaris (L70.0); Hidradenitis suppurativa (L73.2); Vitiligo (L80); Alopecia areata (L63); Lichen planus (L43); Depressive episode (F32); Major depressive disorder (F33) | Any instance of Group 2B occurred on or before the first instance of Group 2A |
| **Non-stress controls** | Group 1A | Index event: general health examination encounter | ICD-10-CM Z00 – Encounter for general examination without complaint or suspected diagnosis | Event between 1 Jan 2015 and 31 Dec 2018; age ≥18 years |
|  | Group 1B | Exclusion of psychological stress diagnoses prior to index | ICD-10-CM F43, Z73.3 | Any instance of Group 1B occurred on or before the first instance of Group 1A |
|  | Group 2A | Index event replication used for cohort construction | Same as Group 1A | Same date window |
|  | Group 2B | Exclusion criteria prior to cohort entry | Same exclusions as listed for cases | Any instance of Group 2B occurred on or before the first instance of Group 2A |

| **S6 (alternative index definition; chronic stress)** | | | | |
| --- | --- | --- | --- | --- |
| **Cohort** | **Query group** | **Definition** | **Codes** | **Temporal rule** |
| **Psychological stress (cases)** | Group 1 | Index event: first recorded diagnosis of psychological stress | ICD-10-CM F43 – Reaction to severe stress and adjustment disorders; Z73.3 – Stress, not elsewhere classified | Event between 1 Jan 2015 and 31 Dec 2018; age ≥18 years |
|  | Group 2A | Index event replication used for cohort construction | Same as Group 1 | Same date window |
|  | Group 2B | Exclusion criteria prior to cohort entry | Burns/corrosions (T20–T25); Presbycusis (H91.1); Appendectomy (CPT 1014622, 44950, 44970); Rosacea (L71); Psoriasis vulgaris (L40.0); Atopic dermatitis (L20); Acne vulgaris (L70.0); Hidradenitis suppurativa (L73.2); Vitiligo (L80); Alopecia areata (L63); Lichen planus (L43); Depressive episode (F32); Major depressive disorder (F33) | Any instance of Group 2B occurred on or before the first instance of Group 2A |
|  | Group 3A | Index event replication used for temporal anchoring | Same as Group 1 | Same date window |
|  | Group 3B | Repeated stress diagnosis confirming exposure persistence | ICD-10-CM F43, Z73.3 | Any instance of Group 3B occurred within 3 months and 3 years after the first instance of Group 3A |
| **Non-stress controls** | Group 1A | Index event: general health examination encounter | ICD-10-CM Z00 – Encounter for general examination without complaint or suspected diagnosis | Event between 1 Jan 2015 and 31 Dec 2018; age ≥18 years |
|  | Group 1B | Exclusion of psychological stress diagnoses prior to index | ICD-10-CM F43, Z73.3 | Any instance of Group 1B occurred on or before the first instance of Group 1A |
|  | Group 2A | Index event replication used for cohort construction | Same as Group 1A | Same date window |
|  | Group 2B | Exclusion criteria prior to cohort entry | Same exclusions as listed for cases | Any instance of Group 2B occurred on or before the first instance of Group 2A |
